# Perilla Seed Meal Extract Enriched with Rosmarinic Acid and Luteolin: Natural Active Pharmaceutical Ingredients (NAPIs) for Osteoprotective Effects

**DOI:** 10.3390/antiox14080973

**Published:** 2025-08-08

**Authors:** Thanawat Pattananandecha, Sutasinee Apichai, Treethip Sukkho, Jetsada Ruangsuriya, Fumihiko Ogata, Naohito Kawasaki, Chalermpong Saenjum

**Affiliations:** 1 Office of Research Administration, Chiang Mai University, Chiang Mai 50200, Thailand; thanawat.patt@cmu.ac.th (T.P.); sutasinee.apichai@cmu.ac.th (S.A.); 2 Research Center for Innovation in Analytical Science and Technology for Biodiversity-Based Economic and Society (I-ANALY-S-T_B.BES-CMU), Chiang Mai University, Chiang Mai 50200, Thailand; treethip.sk@gmail.com (T.S.); jetsada.ruang@cmu.ac.th (J.R.); 3 Department of Biochemistry, Faculty of Medicine, Chiang Mai University, Chiang Mai 50200, Thailand; 4 Faculty of Pharmacy, Kindai University, 3-4-1 Kowakae, Higashi-Osaka, Osaka 577-8502, Japan; ogata@phar.kindai.ac.jp (F.O.); kawasaki@phar.kindai.ac.jp (N.K.); 5 Antiaging Center, Kindai University, 3-4-1 Kowakae, Higashi-Osaka, Osaka 577-8502, Japan; 6 Department of Pharmaceutical Sciences, Faculty of Pharmacy, Chiang Mai University, Chiang Mai 50200, Thailand

**Keywords:** perilla seed meal, functional ingredient, rosmarinic acid, luteolin, osteoporosis

## Abstract

Perilla seed meal (PSM) is a waste biomass of perilla seed extraction that retains flavonoid and phenolic compounds. In this study, we aimed to investigate the potential of PSM extracts (PSMEs) from *Perilla frutescens* (L.) Britton as a sustainable source of natural active pharmaceutical ingredients (NAPIs) containing rosmarinic acid and luteolin for promoting bone health. PSMEs were obtained through shaking incubation and ultrasonic extraction, with 40% ethanol (PS-E40) and 80% ethanol (PS-E80) being found to be the most effective solvents. The effects of PSMEs on bone formation markers were evaluated in human fetal osteoblast cells (hFOB 1.19) using bone formation parameters. The results demonstrated that PS-E40 and PS-E80 extracts significantly increased alkaline phosphatase (ALP) activity, osteocalcin (OC) production, and osteoprotegerin (OPG) levels while concurrently reducing receptor activator of nuclear factor kappa-Β ligand (RANKL) and reactive oxygen species (ROS) production in a dose-dependent manner, particularly at 100 µg/mL on day 7 and 50 and 100 µg/mL on day 14 of the co-incubation period. Moreover, Alizarin Red S staining demonstrated that PS-E40 enhanced calcium deposition in both normal and osteogenic media, further supporting the effect of PSMEs on mineralization and osteoblast differentiation. Our findings suggest that PSMEs rich in rosmarinic acid and luteolin enhance bone health by promoting osteoblast activity and reducing osteoclastogenesis.

## 1. Introduction

Osteoporosis is a multifactorial skeletal disorder characterized by compromised bone strength and increased fracture risk [1,2,3]. It is a major global health challenge, particularly among aging populations, including those in Thailand [4]. The aging population is facing an increasing risk of osteoporosis, which exacerbates issues related to healthcare and the financial burden of treatment [5]. Currently available treatments often involve medications that can cause significant side effects [6,7,8], leading to increased interest in alternative or complementary therapies. In recent years, there has been increasing interest in using natural active pharmaceutical ingredients (NAPIs) as possible treatments for osteoporosis. These NAPIs, including flavonoids and phenolic compounds, have shown potential in promoting bone health through their anti-inflammatory, antioxidant, anti-cancer, and bone resorption-inhibiting activities [9,10,11,12,13].

*Perilla frutescens* (L.) Britton (PF), commonly known as perilla, thrives in the tropical climate of Thailand. It is widely cultivated throughout the country and has both culinary and medicinal value, with it contributing significantly to the local agricultural economy [14,15]. The results of recent studies also highlight perilla as a promising source of flavonoids and other bioactive compounds with potential therapeutic applications [16,17,18]. The main bioactive compounds found in PF include luteolin, apigenin, rosmarinic acid, and chrysoeriol [19], all of which have shown various health benefits in different studies [16,19,20,21].

Rosmarinic acid is a naturally occurring polyphenol that is present in perilla (*Perilla* spp.) and other plants, including Lamiaceae herbs such as rosemary (*Rosmarinus officinalis*), sage (*Salvia officinalis*), and mint (*Mentha* spp.) [22]. It is known for its potent antioxidant, anti-inflammatory, and antimicrobial properties [22,23]. Luteolin is a natural flavonoid [24], found in many fruits, vegetables, and herbs, including perilla [13]. The results of studies on PF extracts indicate that they may help modulate bone formation markers in cell-based experiments [25,26]. Bone formation markers such as alkaline phosphatase (ALP), osteocalcin (OC), osteoprotegerin (OPG), and receptor activator of nuclear factor kappa-Β ligand (RANKL) play crucial roles in bone metabolism and act as markers of osteoblast activity [27]. Studying the impact of these markers derived from NAPIs will therefore provide valuable insights into its mechanism of action in promoting bone formation and remodeling.

Perilla seeds (PSs) are a rich source of bioactive compounds with a variety of pharmacological properties, including anti-allergic, anti-bacterial, anti-inflammatory, hypolipidemic, antioxidant, and anti-cancer effects [16,17,19,28]. Following PS oil extraction, a significant amount of perilla seed meal (PSM) remains. This by-product is a valuable source of nutrients, containing high levels of protein (35–45%), fiber (55–65%), and lower amounts of polysaccharides, polyols, fatty acids, phytosterols, flavonoids, and phenolics [29]. PSM is commonly used as animal feed, representing a cost-effective and nutritious alternative in the industry [29,30]. However, the concept of zero-waste management opens up new avenues for using PSM as an NAPI in medical and health-related applications. PSM can be used to optimize resource efficiency and reduce waste in the production of pharmaceuticals and cosmeceuticals, supporting sustainable practices in the industry.

In this study, we aimed to extract NAPIs from PSM derived from PF and investigate their impact on bone formation markers in human osteoblast cells. We focus on sustainable resource management and waste reduction, exploring the potential of PSM as a bioactive-rich ingredient for bone health by repurposing this agricultural by-product and offering innovative solutions for osteoporosis.

## 2. Materials and Methods

### 2.1. Reagents and Chemicals

High-performance liquid chromatography (HPLC)-grade methanol (LC1115) and acetonitrile (LC1005) and analytical-grade ethanol (AR1380) and sodium hydroxide (AR1171) were purchased from RCI Labscan Limited (Bangkok, Thailand). Standards for rosmarinic acid (R4033), luteolin (L9283), and apigenin (10798) were obtained from Sigma-Aldrich (St. Louis, MO, USA), and quercetin (P0042) and kaempferol (K0018) were purchased from Tokyo Chemical Industry Co., Ltd. (Tokyo, Japan). L-ascorbic acid (A4403), β-glycerophosphate (G5422), Alizarin Red S (K48040178), acetic acid (K53439863-121), 2′,7′-dichlorodihydrofluorescein diacetate (DCFH_2_-DA; D6883), N-acetyl cysteine (NAC; A9165), Bradford Reagent (B6916), bovine serum albumin (BSA) standard (A9418), and CellLytic™ M (C2978) were obtained from Sigma-Aldrich (St. Louis, MO, USA). Dulbecco’s Modified Eagle Medium (DMEM)/F-12, no phenol red (11039021), Geneticin Selective Antibiotic (G418 Sulfate; 10131035), fetal bovine serum (FBS; A5256701), trypsin–EDTA solution (15400054), phosphate-buffered saline (PBS) pH 7.4 (10010023), and PrestoBlue™ Cell Viability Reagents (A13261) were purchased from Thermo Fisher Scientific (Waltham, MA, USA). The Human Osteocalcin ELISA Kit (AB270202), Human Osteoprotegerin ELISA Kit (AB189580), and Human TNFSF11 ELISA Kit (RANKL) (AB213841) were purchased from Abcam (Cambridge, UK). p-nitrophenyl phosphate (pNPP; A12310) was purchased from Alfa Aesar (Haverhill, MA, USA). 4-nitrophenol (pNP; 73560) was purchased from Fluka (Buchs, Switzerland). Dexamethasone (1A 1347/30) was purchased from L.B.S. Laboratory Co. Ltd. (Bangkok, Thailand). Diethanolamine (00098) was purchased from Loba Chemie Pvt. Ltd. (Mumbai, India).

### 2.2. Plant Extraction

The PSM was obtained from the community enterprise of the Pong Yang Ma farmer group, located in Ban Pong Subdistrict, Hang Dong District, Chiang Mai Province, Thailand. It was dried at 100 °C for 3 h to reduce the moisture content and subsequently ground with a blender and sifted through a number 20 sieve, followed by extraction using two methods, shaking incubation (60 °C for 1 h at 140 rpm) and ultrasonic extraction (0–10 °C for 20 min), using the modified methods of Selvakumar Periyasamy and Sivashanmugam [31] and Oroian et al. [32], respectively. Deionized water (PS-DW) and ethanol at various concentrations, 20% (PS-E20), 40% (PS-E40), 60% (PS-E60), and 80% (PS-E80), and absolute ethanol (PS-E100) were used as extraction solvents with the PSM at a solvent ratio of 1:10 (by volume). Upon completion of the extraction process, the extracted solutions were filtered through filter paper (Whatman No. 1). Thereafter, the obtained solvent was removed under low pressure and dried under vacuum. Subsequently, the obtained dried extracts were stored at −20 °C for further analysis.

### 2.3. Flavonoid and Phenolic Compound Determination

Flavonoids and phenolic compounds in PSMEs were determined using HPLC (Agilent Technologies, Inc., Santa Clara, CA, USA) [33]. Briefly, an Agilent 1200 equipped with multiwavelength detection was used for the reverse-phase HPLC system. HPLC was performed using a Symmetry^®^ C18 column (4.6 mm × 250 mm, 5 µm particle diameter, Waters Co., Ltd., Milford, MA, USA). The 1 mL/min of 30% acetonitrile in 0.1% glacial acetic acid served as the mobile phase, with the column temperature set to 30 °C. The sample volume of 10 µL was injected and detected at 350 nm. Luteolin, quercetin, rosmarinic acid, kaempferol, and apigenin were used as standards. To analyze the samples, 10 mg of dry extract was dissolved in 1 mL of methanol and subsequently filtered through 0.22 µm syringe filters. In addition, the mass spectrometry (MS) experiment was conducted using a time-of-flight mass spectrometer. The instrument operated in both positive ion mode and negative ion mode with an ESI capillary voltage of 3.5 V, a desolvation line temperature of 400 °C, a source temperature of 150 °C, and a collision energy of 22 volts. All samples were analyzed in triplicate.

### 2.4. Cell Culture and Cytotoxicity Test

The DMEM/Ham’s F-12 medium supplemented with 10% FBS; 0.3 mg/mL Geneticin Selective Antibiotic was used to culture primary human fetal osteoblast cells (hFOB 1.19, ATCC, Manassas, VA, USA, CRL-11372™; CVCL_3708) under 5% CO_2_ at 37 °C. To determine the cytotoxicity, the cells (1 × 10^4^ cells) were seeded into each well of a 96-well plate and incubated with or without the culture medium containing 25, 50, 100, and 200 µg/mL of the selected PSMEs for 72 h. Subsequently, cytotoxicity was assessed using PrestoBlue™ Cell Viability Reagents.

### 2.5. Effect of PSMEs on Intracellular ROS Production

DCFH_2_-DA was used to monitor ROS production following the method described by Phromnoi et al. [25]. Initially, hFOB 1.19 cells were seeded in a 96-well plate and pre-treated with 10, 25, 50, and 100 µg/mL of the tested samples for 24 h. Next, 50 µM of NAC or L-ascorbic acid (2.5, 5.0, 10, and 25 μg/mL) was used as a positive control. Following treatment with hydrogen peroxide (H_2_O_2_) for 1 h to stimulate ROS production, the 40 µM DCFH-DA solution was subsequently added, and fluorescence was measured after 30 min of incubation. The wavelengths of excitation at 480 nm and emission at 525 nm were set to measure the green fluorescence intensity.

### 2.6. Effect of PSMEs on Bone Formation Parameters

The potential of PSMEs to stimulate bone formation was evaluated using hFOB 1.19 cells. The concentrations of PSMEs that exhibited no cytotoxic effects were selected for this study. The cells (1.0 × 10^4^ cells/well) were seeded in 24-well plates and treated with PSMEs for 7 and 14 days under 5% CO_2_ at 37 °C. Cell lysates were obtained using Cellytic™, and culture medium supernatants were collected for the determination of OC, OPG, and RANKL.

### 2.7. Alkaline Phosphatase (ALP) Activity

The method described by Sukkho et al. [34] was used to determine ALP activity. Briefly, the collected cell lysates were measured by adding pNPP to diethanolamine buffer (pH 9.8) and incubating them at 37 °C for 120 min. The NaOH solution was added to stop the reaction, and the absorbance at 405 nm was subsequently measured to determine ALP activity. ALP activity was measured by hydrolyzing pNPP to pNP, which produces a yellow color. Lastly, the protein concentration was measured using Bradford reagent.

### 2.8. Alizarin Red S Staining

Alizarin Red S staining for mineralization was conducted on hFOB 1.19 cells using a modified version of Bernar et al.’s method [35]. Briefly, cells were seeded at 1.0 × 10^4^ cells/cm^2^ in DMEM/F-12 medium and incubated for 24 h. The medium was then replaced with DMEM/F-12 or osteogenic medium (DMEM/F-12 with 10 mM β-glycerophosphate, 0.01 µM dexamethasone, and 0.2 mM L-ascorbic acid) containing 25 µg/mL of the selected PSME (PS-E40) with the highest ALP activity, and cells were incubated for 7 or 14 days, with medium changes every 3 days. After incubation, cells were fixed with 50% ethanol for 15 min at 4 °C, rinsed with deionized water, and stained with Alizarin Red S solution for 30 min. Calcium deposits were observed under an inverted microscope (Motic AE2000, Kowloon, Hong Kong), and images were captured. The dye was extracted using an extraction solvent (10% acetic acid in deionized water and methanol, 8:2 *v*/*v*), and absorbance was measured at 405 nm using a microplate reader (SpectraMax M3, San Jose, CA, USA).

### 2.9. Osteocalcin (OC), Osteoprotegerin (OPG), and Receptor Activator of Nuclear Factor Kappa Ligand (RANKL) Determination

OC, OPG, and RANKL are key markers associated with bone formation. SimpleStep ELISA^®^ kits, including the human Osteocalcin ELISA Kit, the human Osteoprotegerin ELISA Kit, and the human TNFSF11ELISA Kit (RANKL), were used to determine their levels in the culture medium supernatants by following the manufacturer’s protocols. Briefly, OC and OPG were incubated for 1 h 30 min, whereas RANKL was incubated for 3 h 30 min. The samples were then measured at 450 nm, and the OC, OPG, and RANKL levels were calculated by comparing them to their standard curve and expressed as pg/mL, ng/mL, and ng/mL, respectively.

### 2.10. Statistical Analysis

Data were analyzed using SPSS version 22.0 (IBM Corporation, Armonk, NY, USA) and GraphPad Prism version 9.0 (GraphPad Software, San Diego, CA, USA). One-way ANOVA followed by Tukey’s HSD post hoc test was used to determine significant differences among multiple groups. For comparisons between treated and control groups under specific conditions, two-way ANOVA followed by Dunnett’s post hoc test was applied. Data are expressed as the mean ± standard deviation (SD) from three independent experiments. Statistical significance was defined as * *p* < 0.05, ** *p* < 0.01, *** *p* < 0.001, and **** *p* < 0.0001.

## 3. Results

### 3.1. Flavonoid and Phenolic Compound Content

**Figure 1:**
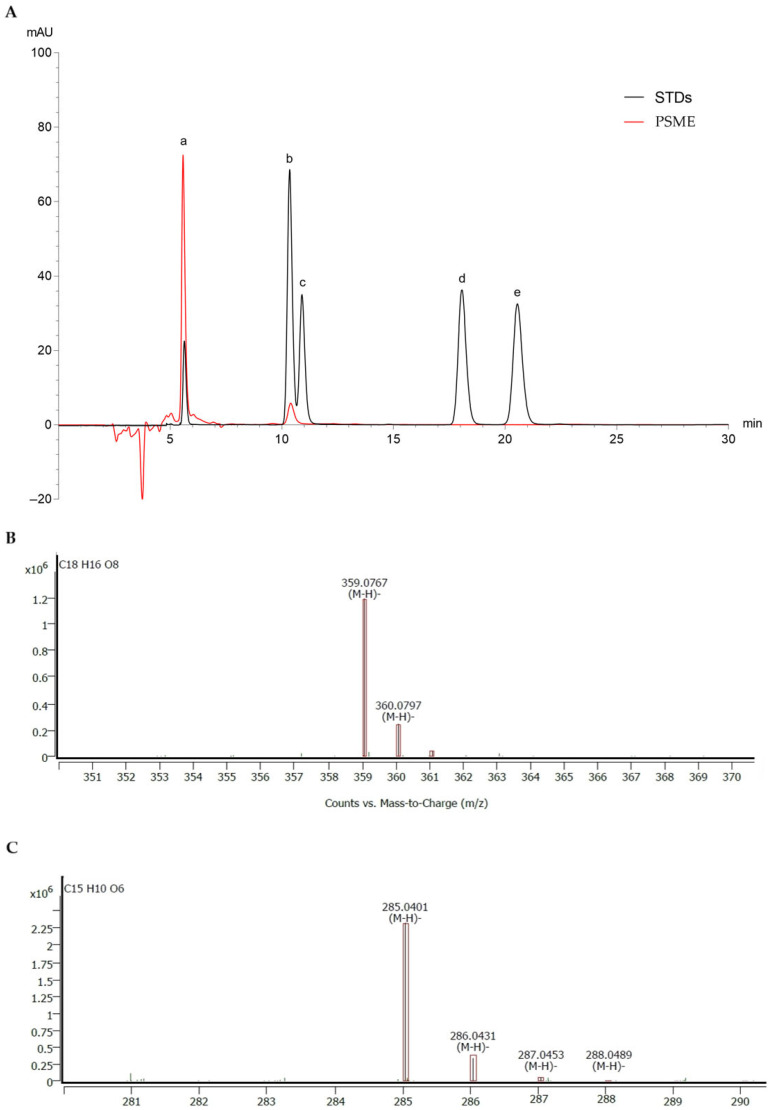
(**A**) HPLC chromatogram of flavonoid and phenolic compound standards, including (a) rosmarinic acid, (b) luteolin, (c) quercetin, (d) apigenin, (e) kaempferol, and the PSME sample. (**B**) MS spectrum of rosmarinic acid and (**C**) MS spectrum of luteolin.

PSMEs were analyzed using HPLC and MS. Rosmarinic acid and luteolin were identified as the major constituents, as shown in Table 1. Their identification was further confirmed by comparison with corresponding standards, as illustrated in the chromatogram and MS spectra (Figure 1). The results indicated that the highest concentration of rosmarinic acid was obtained using 40% ethanol (PS-E40) as the extraction solvent in both the shaking incubation and ultrasonic extraction methods. These concentrations were significantly greater than those obtained with other solvents (*p* < 0.05). The highest concentration (*p* < 0.05) of luteolin was found when using 80% ethanol (PS-E80) as the solvent in both the shaking incubation and ultrasonic extraction methods. In addition, at the same extraction solvent concentration, the shaking incubation method yielded higher amounts of rosmarinic acid and luteolin than the ultrasonic extraction method. PS-E40 and PS-E80 from the shaking incubation method were therefore selected for further analysis, as they had the highest rosmarinic acid and luteolin content, respectively.

### 3.2. Effect of PSMEs on Cell Viability of hFOB 1.19 Cells

**Figure 2:**
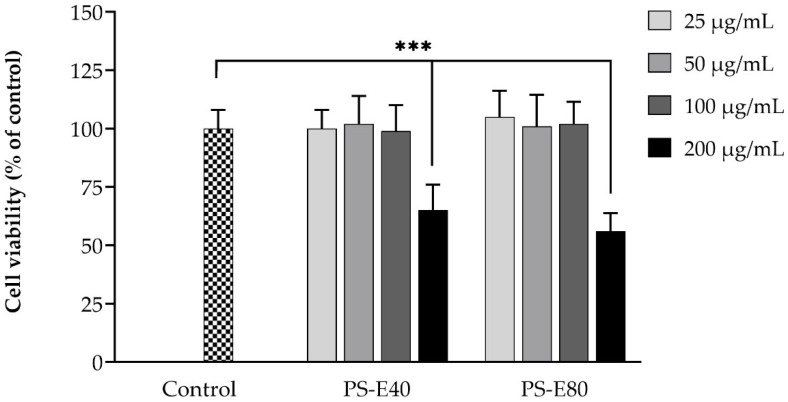
Effect of PSMEs on cell viability of hFOB 1.19 cells. The data are presented as the mean ± SD (n = 3). *** *p* < 0.001 compared to the control.

The cytotoxic effect of the selected PSMEs, PS-E40 and PS-E80 (25–200 µg/mL), on hFOB 1.19 cell viability was determined, as shown in Figure 2. No significant cytotoxicity was observed at concentrations lower than 100 µg/mL. The PS-E40 and PS-E80 concentrations at 25–100 µg/mL were thus selected to study their effects on bone formation in hFOB 1.19 cells.

### 3.3. Effect of PSMEs on Intracellular ROS Production

**Figure 3:**
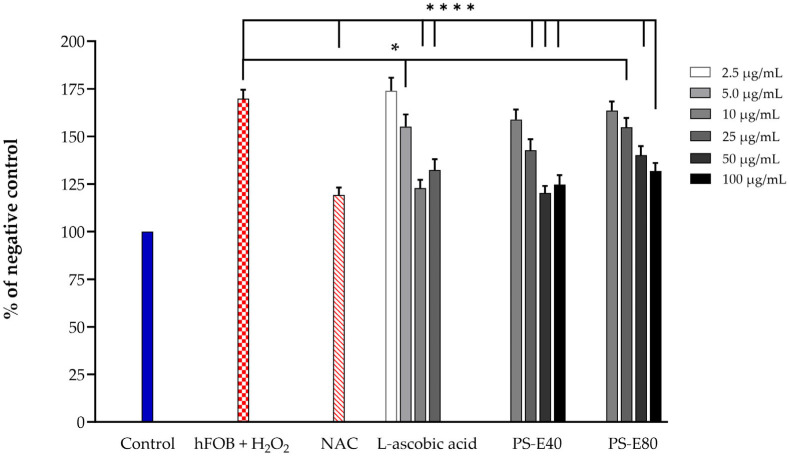
Effect of PSMEs on intracellular ROS production. The data are presented as the mean ± SD (n = 3). * *p* < 0.05 and **** *p* < 0.0001 compared to the hFOB + H_2_O_2_ group.

Exposure of hFOB 1.19 cells to H_2_O_2_ significantly elevated intracellular ROS levels compared to untreated control cells. Treatment with both PS-E40 and PS-E80 at 25, 50, and 100 µg/mL significantly attenuated H_2_O_2_-induced ROS production (*p* < 0.05 to *p* < 0.0001). The antioxidant reference compound, NAC (50 µM), effectively suppressed ROS accumulation (*p* < 0.0001), evidencing the validity of the experimental system. L-ascorbic acid exhibited a concentration-dependent effect, with a significant ROS reduction observed at 10 and 25 µg/mL (*p* < 0.05 and *p* < 0.0001, respectively); in comparison, higher concentrations were less effective (Figure 3).

### 3.4. Effect of Perilla Seed Meal Extracts (PSMEs) on Bone Formation Parameters

#### 3.4.1. Effect of PSMEs on ALP in hFOB 1.19 Cells

**Figure 4:**
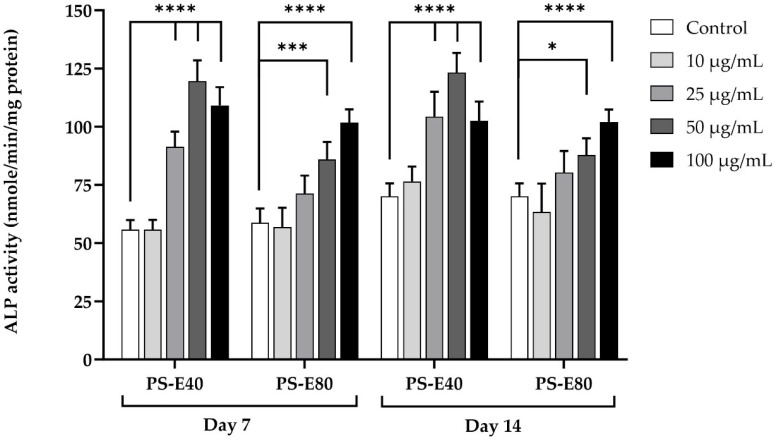
Effect of PSMEs on the alkaline phosphatase activity of hFOB 1.19 cells. The data are presented as the mean ± SD (n = 3). * *p* < 0.05, *** *p* < 0.001, and **** *p* < 0.0001 compared to the control.

PSMEs, including PS-E40 and PS-E80, were able to stimulate the activity of ALP, a biomarker of osteoblast formation, when co-cultured with hFOB 1.19 cells for both 7 and 14 days, as shown in Figure 4. PS-E40 exhibited higher ALP activity than PS-E80 at the same concentration. PS-E40 at concentrations of 25–100 µg/mL and PS-E80 at concentrations of 50 and 100 µg/mL significantly increased ALP activity in comparison with the control group when co-cultured for 7 and 14 days.

#### 3.4.2. Alizarin Red S Staining

**Figure 5:**
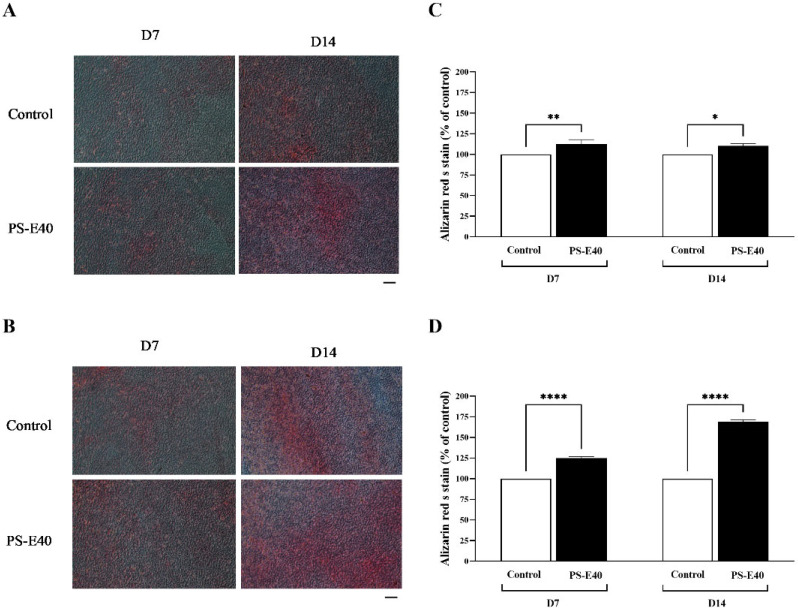
Images of Alizarin Red S staining of hFOB 1.19 cells (scale bar of 100 µm) with and without PS-40E at 50 µg/mL on day 7 and day 14 of incubation in (**A**) DMEM/F-12 medium and (**B**) osteogenic medium and the quantitative analysis of mineralization in hFOB 1.19 cells (mean ± SD, n = 3) with and without PS-E40 at 50 µg/mL on day 7 and day 14 of incubation in (**C**) DMEM/F-12 medium and (**D**) osteogenic medium. * *p* < 0.05, ** *p* < 0.01 and **** *p* < 0.0001 compared to the control.

The mineralization of hFOB 1.19 cells treated with or without PS-E40 (50 µg/mL) was assessed using Alizarin Red S staining on day 7 and day 14 of incubation in both DMEM/F-12 medium and osteogenic medium. The Alizarin Red S staining results demonstrated that the PS-E40-treated cells exhibited greater calcium deposition than the control group in both media, as demonstrated in Figure 5A,B. In addition, the PS-E40-treated group showed more pronounced staining on day 14. Quantitative analysis of the Alizarin Red S staining results presented in Figure 5C,D demonstrates significant differences between the control and PS-E40-treated cells in the DMEM/F-12 medium and osteogenic medium, respectively. In the DMEM/F-12 medium, PS-E40-treated cells exhibited significantly increased mineralization compared to the control on day 7 (*p* < 0.01) and day 14 (*p* < 0.05). In the osteogenic medium, PS-E40-treated cells also showed a highly significant increase in mineralization on both day 7 and day 14 (*p* < 0.0001).

#### 3.4.3. Effects of PSMEs on OC, OPG, and RANKL Levels in hFOB 1.19 Cells

**Figure 6:**
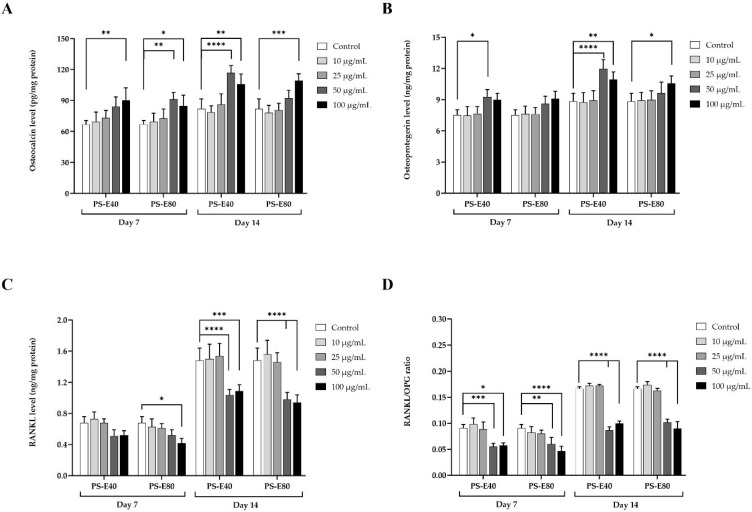
Effect of PSMEs on (**A**) osteocalcin, (**B**) osteoprotegerin, and (**C**) RANKL levels and (**D**) the RANKL/OPG ratio in hFOB 1.19 cells. The data are presented as the mean ± SD (n = 3). * *p* < 0.05, ** *p* < 0.01, *** *p* < 0.001, and **** *p* < 0.0001 compared to the control.

The production of OC by hFOB 1.19 cells is illustrated in Figure 6A. The results indicated that PS-E40 at a concentration of 50 µg/mL and PS-E80 at concentrations of 50 and 100 µg/mL had a significant effect on OC production on day 7. On day 14, PS-E40 at concentrations of 50 and 100 µg/mL and PS-E80 at 100 µg/mL significantly increased OC production compared to the control. As shown in Figure 6B, the production of OPG in hFOB 1.19 cells was significantly increased by PS-E40 treatment at a concentration of 50 µg/mL after 7 days and at concentrations of 50 and 100 µg/mL after 14 days of co-culture compared to the control. Similarly, PS-E80 treatment at a concentration of 100 µg/mL after 14 days significantly increased OPG production compared to the control. RANKL, a key regulator of osteoclast formation and bone resorption, was assessed to determine the effects of PSMEs on bone resorption. The results (Figure 6C) demonstrated that RANKL levels were lower on day 7 compared to day 14. The cells treated with PS-E40 at concentrations of 50 and 100 µg/mL significantly inhibited RANKL production on day 14. In comparison, PS-E80 at 100 µg/mL after 7 days and 50 and 100 µg/mL after 14 days significantly reduced RANKL levels compared to the control. The results presented in Figure 6D demonstrate the effect of PS-E40 and PS-E80 on the RANKL/OPG ratio in hFOB 1.19 cells. Both extracts at 50 µg/mL and 100 µg/mL significantly lowered the RANKL/OPG ratio compared to the control, both on day 7 and day 14.

## 4. Discussion

As part of the present study, we aimed to investigate the effects of PSMEs on bone formation markers, including ALP, calcium deposition using Alizarin Red S staining, OC, OPG, and RANKL, in osteoblast cell lines, focusing on rosmarinic acid and luteolin as primary components. Optimizing the extraction process requires an understanding of the relationship between target compound polarity and solvent selection. The results of recent studies indicate that the solubility of phenolic compounds is significantly influenced by the solvent polarity and the chemical composition of the plant material [36,37]. Our results demonstrate the impact of solvent polarity on the extracted flavonoid and phenolic acid levels. Based on our HPLC analysis results, 40% ethanol (PS-E40) extraction yielded significantly higher rosmarinic acid concentrations; in comparison, 80% ethanol (PS-E80) yielded a higher concentration of luteolin. This result aligns with the differing polarities of these compounds, as indicated by their Log *p* values: rosmarinic acid (Log P ≈ 1.70) [38] exhibits greater polarity than luteolin (Log P ≈ 2.53) [39]. The higher polarity of rosmarinic acid makes it more soluble in 40% ethanol; in comparison, the lower polarity of luteolin makes it suitable for the extraction of 80% ethanol. In addition, the shaking incubation method proved to be a more suitable approach for semi-industrial or industrial production, in addition to producing a higher concentration of active compounds than ultrasonic extraction. The gentler conditions of shaking incubation facilitate more efficient solvent penetration, preserve compound integrity, and enable more selective extraction compared to the intense energy input of ultrasonic extraction. Consequently, PS-E40 and PS-E80 extracts obtained using the shaking incubation method were selected for further analysis.

In the cell viability test, PS-E40 and PS-E80 extracts demonstrated no significant cytotoxicity below 100 µg/mL. The rise in the cytotoxic effect at a higher PSME concentration (200 µg/mL) could be induced by other compounds present in the extract. Thus, to ensure safety, the concentrations of 25–100 µg/mL were used in the further investigation of hFOB 1.19 cells. PSMEs significantly reduced H_2_O_2_-induced intracellular ROS levels in hFOB 1.19 cells, suggesting their antioxidant potential in osteoblasts. This effect is likely due to the presence of rosmarinic acid and luteolin, the major active compounds enriched in PS-E40 and PS-E80, respectively. Rosmarinic acid has been reported to reduce oxidative stress by enhancing antioxidant enzyme activities, such as SOD, CAT, and GSH-Px, and suppressing the PI3K/AKT signaling pathway, thereby protecting cells from oxidative injury [40,41]. Luteolin similarly contributes by scavenging ROS, upregulating antioxidant enzymes, and protecting cells from oxidative damage. Furthermore, luteolin has been shown to attenuate H_2_O_2_-induced apoptosis, restore glutathione levels, and modulate signaling pathways involved in cell survival and inflammation [42,43].

The effects of PSMEs on bone formation markers were investigated in hFOB 1.19 cells. The results clearly demonstrated that PSMEs, both PS-E40 and PS-E80, stimulated ALP activity in hFOB 1.19 cells. ALP is an enzyme produced by early-stage osteoblasts, and it is considered a key early marker of osteoblast differentiation and bone formation [44]. It plays a crucial role in mineralizing the bone matrix by hydrolyzing pyrophosphate, a molecule that inhibits mineralization [45,46]. PS-E40 and PS-E80 treatment increased ALP activity, which suggested that these extracts could promote osteoblast differentiation and subsequent bone formation. Notably, PS-E40, which possesses a higher concentration of rosmarinic acid, exhibited a more pronounced effect. Furthermore, PS-E40, the extract selected for calcium deposition analysis, significantly enhanced mineralization in hFOB 1.19 cells. This effect was observed under both standard (DMEM/F-12) and osteogenic conditions, with a more pronounced impact in the osteogenic medium. PS-E40 significantly increased calcium deposition in standard and osteogenic conditions, indicating that it promotes mineralization, with the osteogenic medium further enhancing this effect. This finding aligns with the literature findings on rosmarinic acid, which has been reported to promote osteoblast differentiation and bone formation through mechanisms such as the activation of the Wnt/β-catenin signaling pathway [47]. The results of a study involving MC3T3-E1 cells cultured on titanium surfaces demonstrated that rosmarinic acid treatment increased ALP activity and mineralization, as confirmed by Alizarin Red S staining [48]. Beyond rosmarinic acid, luteolin, also abundant in PSMEs, plays a crucial role. Based on the findings of Choi [49], luteolin significantly increased collagen content and ALP activity in MC3T3-E1 osteoblastic cells and reduced oxidative stress and levels of inflammatory cytokines such as TNF-α and IL-6. Its osteogenic effects extend to human periodontal ligament cells (HPDLCs), where luteolin has been shown to promote osteogenic differentiation by increasing the expression of bone morphogenetic protein 2 (BMP2), OC, runt-related transcription factor 2 (RUNX2), Osterix (OSX), β-catenin, and cyclin D1, primarily through activation of the Wnt/β-catenin signaling pathway [50].

OC is a protein secreted by mature osteoblasts and embedded in the bone matrix that plays a critical role in bone formation and mineralization [51,52]. It regulates the deposition of hydroxyapatite crystals in the bone matrix, which increases bone density and strength [53]. In our study, OC levels significantly increased following treatment with PS-E40 and PS-E80, especially at higher concentrations (50–100 µg/mL) and after prolonged co-culture with the extracts (at 14 days), indicating a time-dependent response. Given that OC is a late marker of osteoblast activity, the delayed increase suggests that prolonged exposure to the extracts may be required to support osteogenic effects fully. These results are consistent with the results of previous studies demonstrating that rosmarinic acid enhances the expression of OC mRNA in MC3T3-E1 cells on titanium surfaces [48]. Similarly, luteolin increased OC secretion in osteoblastic MC3T3-E1 cells and HPDLCs, providing evidence of its role in enhancing bone mineralization and strength [49,50].

OPG acts as a decoy receptor for RANKL, a key molecule that promotes osteoclast formation and bone resorption [54,55]. By blocking RANKL from binding to its receptors on osteoclast precursors, OPG inhibits their differentiation and activity, thereby promoting bone homeostasis and favoring bone formation over resorption [56]. In our study, the increase in OPG levels, particularly with PS-E40, highlights its potential to inhibit osteoclastogenesis. PS-E80 was responsible for the increase but a lower OPG level than PS-E40, possibly due to differences in the concentration or bioavailability of specific bioactive compounds, particularly rosmarinic and luteolin content. Both PS-E40 and PS-E80 significantly reduced RANKL levels; consequently, the RANKL/OPG ratio was significantly lower than the control in a dose-dependent manner, indicating a favorable shift toward bone formation. Our findings align with the existing literature on the major active compounds in PSMEs. Rosmarinic acid has been reported to enhance the osseointegration of osteoblast cells by increasing differentiation, mineralization, and bone formation via the RANKL/RANK/OPG pathway during differentiation in MC3T3-E1 osteoblastic cells on the Ti surface [48]. It further enhances osteoblast activity by activating the canonical Wnt signaling pathway through induced LacZ expression and stabilization of β-catenin in ST2 cells from T-cell factor/β-catenin TOP-GAL mutant mice. In addition, it suppresses osteoclast formation in co-cultures of mouse bone marrow cells and osteoblasts, inhibits RANKL-induced osteoclastic differentiation in bone marrow-derived macrophages, and also reduces RANKL-induced p38 MAPK activation and the expression of NFATc1, c-Jun, and c-Fos in these macrophages [47]. Furthermore, the rosmarinic acid-enriched fraction from PSM has been shown to downregulate RANKL-induced NF-κB, AP-1 activation, and the nuclear factor of activated T-cell 1 (NFATc1) expression, suppress RANKL-induced osteoclast-specific marker gene-like MMP-9, and inhibit RANKL-induced ROS production in RAW 264.7 cells [25]. Luteolin has been reported to inhibit osteoclast formation by blocking RANKL-induced signaling pathways and disrupting the formation of actin rings in mature osteoclasts. This effect directly targets osteoclast progenitors, effectively inhibiting their differentiation without affecting cell viability [57]. This specificity is further demonstrated by luteolin’s ability to selectively regulate key signaling pathways, such as ATF2, p38 MAPK, and NFATc1, which are essential for osteoclast formation. Moreover, luteolin significantly increased OPG levels while concurrently decreasing RANKL levels in rats with periodontitis, suggesting its potential to modulate bone resorption and promote bone formation in periodontal diseases [58]. In addition, increasing OPG and RANKL levels with longer incubation periods provides the extracts’ active compounds with more time to interact with cellular pathways involved in bone remodeling. Prolonged incubation may optimize the compounds’ effect on gene expression and enhance cellular uptake, upregulating pathways that increase RANKL and OPG production. Furthermore, a cumulative effect of PSMEs may be possible due to prolonged exposure, which gradually increases the cellular response.

## 5. Conclusions

In conclusion, the results of this study demonstrate the potential of PSMEs as a sustainable source of NAPIs, rich in rosmarinic acid and luteolin, for promoting bone health. PS-E40 and PS-E80, obtained using the shaking incubation method with 40% and 80% ethanol, respectively, were selected for further analysis as they had the highest rosmarinic acid and luteolin content. Both extracts significantly reduced intracellular ROS levels induced by H_2_O_2_ in hFOB 1.19 cells, indicating potent antioxidant activity comparable to NAC and L-ascorbic acid. By mitigating oxidative stress, PSMEs may indirectly suppress osteoclastogenesis, reinforcing the antioxidant–bone health connection. PS-E40 and PS-E80 exhibited significant beneficial effects on bone biomarkers, including ALP, OC, OPG, and RANKL in hFOB 1.19 cells, indicating their potential to inhibit osteoclastogenesis and promote bone formation. Notably, calcium deposition assessed through Alizarin Red S staining was observed with PS-E40. These osteogenic effects were dose-dependent. These findings provide valuable insights into the mechanisms of action of PSMEs, positioning them as promising candidates for developing therapeutic approaches to osteoporosis and highlighting their potential incorporation into functional foods and nutraceuticals.

## Figures and Tables

**Table 1:** The rosmarinic acid and luteolin content in PSMEs determined using the shaking incubation and ultrasonic extraction methods.

Extraction Solvent	Shaking Incubation		Ultrasonic Extraction	
	Rosmarinic Acid (mg/g Extract)	Luteolin (mg/g Extract)	Rosmarinic Acid (mg/g Extract)	Luteolin (mg/g Extract)
PS-DW	121.90 ± 6.03 ^d^****	1.17 ± 0.05 ^d^	89.46 ± 4.68 ^e^	0.89 ± 0.05 ^f^
PS-E20	169.86 ± 3.00 ^b^****	3.02 ± 0.10 ^c^	130.78 ± 3.14 ^b^	2.71 ± 0.07 ^e^
PS-E40	189.62 ± 2.77 ^a^****	5.03 ± 0.08 ^b^****	152.27 ± 3.40 ^a^	3.56 ± 0.09 ^c^
PS-E60	160.70 ± 3.07 ^b^****	5.61 ± 0.11 ^b^****	135.57 ± 5.31 ^b^	4.12 ± 0.05 ^b^
PS-E80	147.04 ± 2.36 ^c^****	6.90 ± 0.13 ^a^****	117.85 ± 3.06 ^c^	4.98 ± 0.07 ^a^
PS-E100	78.01 ± 3.34 ^e^****	3.93 ± 0.84 ^c^**	47.88 ± 4.31 ^e^	3.04 ± 0.08 ^d^

Data are presented as the mean ± SD (n = 3). Different superscript letters indicate significant differences in compound content between extraction solvents. ** *p* < 0.01 and **** *p* < 0.0001 indicate significant differences in compound content between extraction methods.

## Data Availability

The datasets used and/or analyzed during the current study are available from the corresponding author upon reasonable request.
